# The COMFORT trial: a randomised control trial comparing group-based *COM*passion-*FO*cussed therapy and breathing pattern *ReT*raining with treatment as usual on the psychological functioning of patients diagnosed with cancer recurrence during COVID

**DOI:** 10.1186/s13063-023-07088-4

**Published:** 2023-02-06

**Authors:** Sinead Lynch, Damien Lowry, Clodagh Finnerty, Yvonne O’Meara, Donal Brennan

**Affiliations:** 1 Department of Psychology, Mater Misericordiae University Hospital, Dublin, Ireland; 2 Department of Psycho-Oncology, Mater Misericordiae University Hospital, Dublin, Ireland; 3 Department of Pain Medicine, Mater Misericordiae University Hospital, Dublin, Ireland; 4 COMFORT Trial, University College Dublin, Dublin, Ireland; 5 Women’s Cancer Survivorship Research Coordinator, School of Medicine, University College Dublin, Dublin, Ireland; 6 Mater Misericordiae University Hospital, Dublin, Ireland; 7 School of Medicine, University College Dublin, Dublin, Ireland

**Keywords:** Cancer, Oncology, Recurrence, Compassion-focussed therapy, Radical breathing retraining, Psychological distress, Randomised control trial

## Abstract

**Background:**

A cancer diagnosis is a known precipitant of psychological distress, with fear of recurrence being a well-documented distressing consequence of cancer. Cancer recurrence often results in an additional psychological burden, which may exacerbate as a result of the COVID-19 pandemic.

**Methods:**

This is a single-centre, prospective, randomised controlled trial. Patients identified as having experienced cancer recurrence since March 2020 (the onset of the COVID-19 pandemic in Ireland) will be screened for participation. Eligible, consenting candidates who score 4 or higher on the Distress Thermometer will be enrolled in the study. Participants will be randomly allocated to receive either a 6-week, group-based, online, compassion-focussed therapy and breathing pattern retraining intervention or the control arm. Those in the control arm will all be offered the group intervention after the 18-week study period. The primary outcome is the Distress Thermometer score at 18 weeks post-baseline though secondary outcomes will include measures of mood, traumatic distress and mental adjustment to cancer.

**Discussion:**

To our knowledge, this protocol describes the first RCT which directly examines the effect of a group-based psychological intervention on Irish patients experiencing cancer recurrence in the context of COVID-19. The outcome of this trial is likely to be twofold: It will determine if the psychological intervention achieves its primary objective of distress amelioration 3 months post-intervention and to establish the feasibility of delivering this intervention in a virtual format.

**Trial registration:**

ClinicalTrials.gov NCT05518591. Registered on 25 August 2022. All items from the World Health Organization Trial Registration Data set have been included.

## Administrative information

**Note:** the numbers in curly brackets in this protocol refer to SPIRIT checklist item numbers. The order of the items has been modified to group similar items (see http://www.equator-network.org/reporting-guidelines/spirit-2013-statement-defining-standard-protocol-items-for-clinical-trials/).

**Table:** 

Title {1}	The COMFORT Trial: A randomised control trial comparing group-based COMpassion FOcused Therapy and breathing pattern ReTraining with Treatment As Usual on the psychological functioning of patients diagnosed with cancer recurrence during COVID.
Trial registration {2a and 2b}.	This trial was pre-registered on ClinicalTrials.gov on 25 August 2022. Identifier number: NCT05518591. All items from the World Health Organisation Trial Registration Data set have been included. https://clinicaltrials.gov/ct2/show/ NCT05518591
Protocol version {3}	Protocol version 1: August, 2022
Funding {4}	This trial is being funded by the Irish Cancer Society (Grant code: COV21LYN). University College Dublin is the Host Institution/Sponsor and the Mater Misericordiae University Hospital is the clinical site.
Author details {5a}	(1). Dr Sinead Lynch*, Senior Counselling Psychologist, Depts. of Psychology and Psycho-Oncology, Mater Misericordiae University Hospital. SineadLynch@mater.ie (Correspondence). *Co-first author (2). Dr Damien Lowry*, Chartered Senior Counselling Psychologist, Depts. of Psychology and Pain Medicine, Mater Misericordiae University Hospital, Dublin, Ireland; dlowry@mater.ie (Correspondence). *Co-first author (3). Ms Clodagh Finnerty, Research Assistant, COMFORT trial, University College Dublin. (4). Yvonne O’Meara, Women’s Cancer Survivorship Research Coordinator, School of Medicine, University College Dublin. (5). Prof Donal Brennan, Consultant Gynaecological and Medical Oncologist and Professor of Gynaecological Oncology and Medical Oncology, Mater Misericordiae University Hospital and School of Medicine, University College Dublin.
Name and contact information for the trial sponsor {5b}	The Sponsor for this Study is The School of Medicine, University College Dublin, Ireland. Contact Person: Prof Donal Brennan, Professor of Gynaecological Oncology and Medical Oncology, Email:school.medicine@ucd.ie, Office 00 353 1 716 6603
Role of sponsor {5c}	This is a hypothesis-driven, investigator-initiated trial. Therefore, the funders played no role in the design of the study, data collection, analysis, interpretation of data, or in the writing of the manuscript, other than by way of reviewing the grant application.

## Introduction

### Background and rationale *{6a}*

There are over 170,000 people living with and beyond a cancer diagnosis in Ireland [1]. A cancer diagnosis may create a state of mental anguish that is sometimes more punishing than the physical presence of the disease itself [2]. Fear of recurrence (FoR) is a well-documented, distressing consequence of cancer [3–5]. A diagnosis of recurrent or metastatic disease is regularly associated with reduced psychological well-being, the recovery from which is often slower than after the initial diagnosis [6].

COVID-19 is reported to have initially emerged in Wuhan, China, in late 2019, spreading rapidly and resulting in the global pandemic that has characterised life since early 2020 [7]. It is well-recognised that cancer patients are at an increased risk of experiencing psychological distress compared to the general population as cancer is a concrete threat to life and may deeply modify a person’s social, emotional, and relational world [8]. The COVID-19 pandemic has led to additional psychological distress due to service disruptions, delayed diagnostic tests, surgery and treatments as well as the increased risk of contracting the infection during hospital visits [9–11]. Moreover, COVID-19-related hospital restrictions have led to the reduction or cancellation of visitation rights for inpatients and patients have had to largely attend clinic appointments on their own. Finally, social support from family and community has been less accessible, all of which normally helps to mitigate psychological distress [12].

In 2021, the Mater Misericordiae University Hospital (MMUH) developed a specialised Psycho-Oncology service seeking to address the psychological issues facing patients with cancer diagnoses. Psychological oncology interventions seek to address the psychological, behavioural, medical and social factors that may influence the disease process and the patients’ experience of their care. It recognises that cancer has a significant psychological impact on patients and families and that this can occur at any time from the time of diagnosis onwards. It has been reported that between 20 and 52% of patients show a significant level of psychosocial distress with between 10 and 15% of that group experiencing significant levels of distress [13].

To our knowledge, the psychological distress caused by a cancer recurrence since the onset of the COVID-19 pandemic has not yet been evaluated, though several studies cite a fear of recurrence or disease progression among patients [5, 10, 14, 15]. In this context, we are interested in investigating the potential effect that a psychological intervention might have on patient distress. The National Comprehensive Cancer Network (NCCN) Clinical Practice Guidelines in Oncology advocate the use of the Distress Thermometer (DT) as the screening tool to be used to monitor distress in patients at key points in the cancer experience [13], which is our rationale for using it as our primary outcome measure.

Compassion-focussed therapy (CFT) is an increasingly popular form of psychological therapy and is considered part of the third wave of cognitive behavioural therapies (CBTs). It is an approach that helps people develop and work with experiences of inner warmth and acceptance, self-soothing and self-compassion [16], all of which are relevant to cancer recurrence and COVID-19-related stressors. Existing studies demonstrate promising results across disorders and in transdiagnostic groups [17–21]. In particular, CFT offers promise in targeting the common psychological problems of shame and self-criticism which are common problems in cancer patients with regard to self-treatment [16]. Its holistic and integrative approach to universal human suffering means it is well placed as a therapeutic intervention for cancer patients.

Breathing pattern retraining (BPR) methods activate bodily reactions linked to hyperventilation and can be utilised to facilitate states of relaxation and self-soothing. Some physical sensations from stress and anxiety present as dysfunctional breathing including breathing difficulties, breathlessness and chest pain, symptoms commonly experienced in cancer. Breathlessness is a symptom that features commonly in cancer, particularly in advanced or recurrent disease, and may be linked to breathing dysregulation often posited to be caused by autonomic nervous system disturbances. Drivers of this include fear, stress and anxiety and can lead to autonomic nervous system arousal which leads to an increased ventilatory drive, which in turn leads to altered breathing patterns becoming established and perpetuating the cycle of dysfunctional breathing and anxiety triggers. Given that effective, efficient, responsive and appropriate breathing patterns are influenced by complex interactions between biochemical, biomechanical and cognitive emotional factors, developing a collaborative programme between both physiotherapy and psychology to address all three elements would support its effective management in this patient group. Dysfunctional breathing symptoms can be helped by (i) breathing retraining exercises, (ii) guided relaxation and (iii) mindfulness. We therefore aim to develop an intervention to address physical and psychological symptoms that impact breathing and anxiety [22].

In summary, our combined intervention seeks to address both the psychological and mechanical processes involved in the mediation of distress, within the context of cancer recurrence during the COVID-19 pandemic. This is the basis of our combined approach. We seek to examine whether a combined psychological and mechanical breathing training intervention can achieve a more reductive effect on general distress than the standard treatment, for individuals with a diagnosis of recurrent cancer since March 2020. Should the study demonstrate effective distress amelioration in the intervention arm, it would serve as the basis to roll out the intervention out at a national level to mitigate the effects of COVID-19 and its impact on healthcare in relation to this sizeable patient cohort. It is important to note that the control group (treatment as usual (TAU)) will be offered the opportunity to avail of the therapeutic intervention at a later date after their participation in the 18-week study protocol.

### Objectives *{7}*

The primary objective of this randomised control trial (RCT) is to examine whether a group-based, compassion-focussed therapy (CFT) psychological intervention combined with breathing pattern retraining (BPR), is effective at reducing psychological distress measured using the Distress Thermometer at 3 months following randomisation, in individuals experiencing cancer recurrence during COVID-19, as compared to those receiving TAU.

Secondary objectives of the study include examining whether CFT and BPR demonstrably improve measures of depression, anxiety, trauma symptoms and mental adjustment to cancer, compared to those receiving TAU.

### Trial design *{8}*

**Fig. 1:**
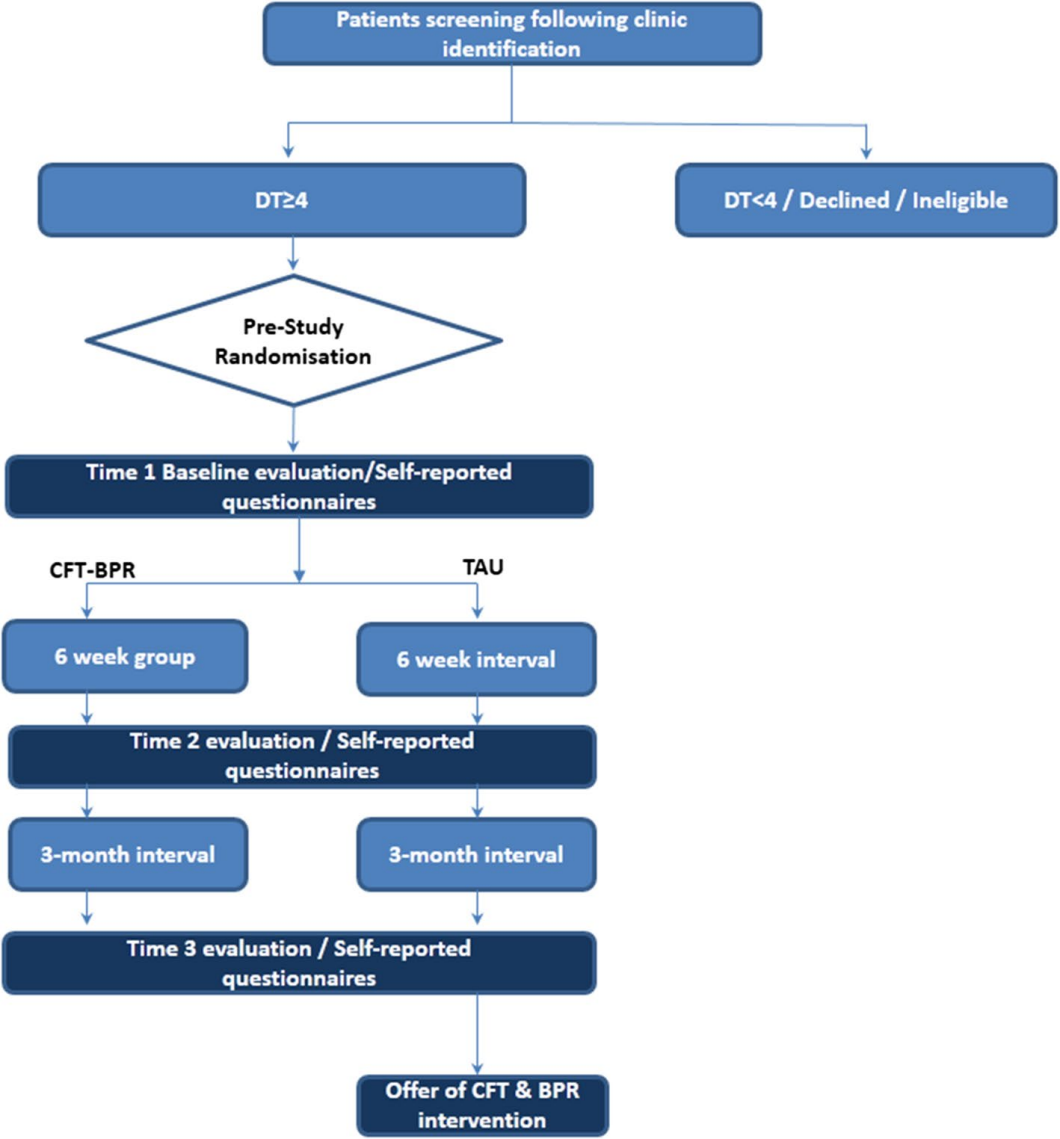
Study flowchart

The COMFORT trial is designed as a prospective, randomised, controlled, single-centre superiority trial with two parallel groups and a primary endpoint of psychological distress 18 weeks post-enrolment. The study will randomise patients who have experienced a cancer recurrence since March 2020 into two groups on a 1:1 basis: one that receives compassion-focussed therapy and radical breathing retraining and the other control group who receives TAU. As all patients will be endorsing elevated levels of distress, it is possible that they will be indicated for individual psychosocial care, as part of the hospital’s psycho-oncology service (CNS, Social Work, Psychology & Psychiatry), but this is likely to be controlled for across both groups. Figure 1 illustrates the study design, timelines of the intervention and baseline as well as follow-up evaluations. The estimated duration for the main investigational plan (e.g. from the start of screening of the first participant to the last participant processed and finishing the study) is 15–18 months.

## Methods: participants, interventions and outcomes

### Study setting *{9}*

This study will be conducted in a tertiary university teaching hospital in Dublin (Mater Misericordiae University Hospital), Republic of Ireland.

### Eligibility criteria *{10}*

Inclusion criteria are:

- Adults aged 18 or older
- Pre-existing cancer diagnosis
- Diagnosis of cancer recurrence during COVID-19 (March 2020 onwards) including a documented recurrent, progressive or metastatic cancer
- Living in Ireland
- English speaking/fluency
- Access to Web/tech support

Exclusion criteria are:

- A score <4 on the Distress Thermometer
- Patient non-consent
- Severe mental illness (e.g. schizophrenia, personality disorder and/or active severe illness)
- Known or suspected drug or alcohol abuse problems within the past 3 months.
- Inability to follow the study procedures, e.g. dementia or non-fluency of English
- Technological inability
- Life expectancy <3 months

### Who will take informed consent? *{26a}*

The research assistant for this trial (CF) will screen for potential study participants from a series of Hospital oncology and haematology outpatient clinics. Nursing colleagues working at these clinics will be assisting in the recruitment process. Once interested candidates meet the eligibility criteria, they will be provided with participant information leaflets, and informed written consent will be obtained by way of in-person clinic attendance. All potential participants will have the opportunity to withdraw at any time point during the study period.

### Additional consent provisions for collection and use of participant data and biological specimens *{26b}*

This is not applicable. No data and/or biological specimens will be collected for use in ancillary studies.

## Interventions

### Explanation for the choice of comparators *{6b}*

Compassion-focussed therapy (CFT) is a holistic and integrative psychological approach to universal human suffering, which makes it well placed as a psychotherapeutic intervention for cancer patients. It is being combined with breathing pattern retraining (BPR), used in Buteyko methods [23], to activate bodily reactions linked to hyperventilation and show patients how to facilitate their own states of relaxation and self-soothing just by breathing in a better way. The Buteyko method, or Buteyko breathing technique, is a form of physical therapy that proposes the use of breathing exercises primarily as a treatment for respiratory conditions, and the principal investigator (SL) has trained in this breathing method. It is being chosen due to reports of individuals with cancer breathing more rapidly throughout the course of cancer treatment. We aim to examine the effectiveness of this combined intervention in reducing distress intensity 3 months post-intervention. To achieve this, we have designed our trial to compare this approach with a TAU control arm.

### Intervention description *{11a}*

#### The intervention

There is one active arm to this study, involving a combined intervention of CFT and BPR, over a 6-week period by way of an online platform. CFT is an integrated and multimodal approach that draws from evolutionary, social, developmental and Buddhist psychology and neuroscience. One of its key concerns is to use compassionate mind training to help people develop and work with experiences of inner warmth, safeness and soothing, via compassion and self-compassion. Compassionate mind training refers to specific activities designed to develop compassionate attributes and skills, particularly those that influence affect regulation. These issues are regularly affected in cancer diagnoses, which makes it a well-placed psychotherapeutic intervention for this patient cohort. The intervention will be delivered by the same psychologist (SL) who works in the specialist area of Psycho-Oncology who has also undergone specialist training in BPR.

#### Intervention group: compassion-focussed therapy and breathing pattern retraining

The CFT and BPR intervention will be delivered in a group format and will last approximately 90 min per group session. Group sessions will each comprise two segments, one being CFT and the other being BPR. The CFT will seek to emphasise concepts such as practising compassion, fostering self-awareness, self-soothing, mindfulness exercises, reducing self-criticism and practising kindness and inner warmth. The BPR segments will seek to introduce and develop breath work that can aid self-soothing, relaxation and bodily activation. The physicality of the breath work is largely performed by the treating psychologist (SL) but is also assisted by a senior physiotherapist from the Mater hospital by way of training videos that participants can view and use for practice. Further details of each individual session and its content are provided in Table 1.

**Table 1:** Layout of the 6-week compassion-focussed therapy and breathing pattern retraining intervention

Session	Summary of sessions
1	Part 1: Breathing pattern retraining education, breathing assessment, simple breath work. Physiotherapy demo in video and simple breath work Part 2: Understanding compassion, CFT explained, model and intervention review; why we need compassion, our tricky brains and defining emotions such as shame, fear and loneliness; Monkey Mind meditation, just noticing our busy minds
2	Part 1: Motivation and our complex brains; understanding motives and emotions—breaking through fear and blocks Part 2: How our thoughts and images affect our minds and brains; breathing patterns—the mind–body connection. Breathing pattern retraining education—exploring the physiotherapy-led exercises
3	Part 1: Compassionate thinking; the 3 components of self-compassion: mindfulness, common humanity and kindness. How this links with soothing breathing Part 2: Dysfunctional breathing and stress; breathing awareness and breathing practices such as tender self-compassion break and affectionate breathing
4	Part 1: Compassionate mind training through imagery—creating your compassionate self Part 2: Creating your compassionate self; breathing pattern retraining techniques and practice combining compassionate imagery
5	Part 1: From self-criticism and loneliness/fear to self-compassion. Distancing and wise observing; breaking identification with one’s thoughts Part 2: Loving kindness meditation; visualising ideal compassionate self; using our compassionate self to work on self-criticism
6	Part 1: Compassionate thinking, attention, behaviour and feeling; cultivating kindness; letter to self and final session Part 2: Looking forward—sustaining our compassionate mind; what I have learned and achieved? Reading letter-to-self using the breath

### Criteria for discontinuing or modifying allocated interventions *{11b}*

Criteria for discontinuing the allocated interventions are:

1. Patient withdrawal of consent at any point of the study
2. Worsening mental health or psychological well-being of the patient such as the onset of severe mental illness as diagnosed by a hospital psychiatrist
3. Significant deterioration of patient’s physical health and/or death

### Strategies to improve adherence to interventions *{11c}*

All participants for this trial will be eligible to receive the psychological and breathing interventions (see the ‘Intervention description {11a}’ section). Those in the treatment arm of the study will be offered the intervention immediately after enrolment and those in the control arm of the study will have the option of completing the group programme once the study period has been completed. Additional measures to aid adherence include its virtual format, ensuring there are no geographical barriers precluding someone from participating. Moreover, the group sessions will only last approximately 90 min, inclusive of a break, to minimise the content burden. All participants will also be well-informed during the consent process. The study participation burden, such as completion of primary outcome questionnaires at t(0) (baseline), t(1) (post-6-week group intervention) and t(2) (3 months post-intervention period), post-enrolment will be explained. The research assistant responsible for screening potential study participants will record all eligible candidates and those that do not proceed with the study due to ineligibility, non-consent or withdrawal. In so far as it is possible, only eligible and fully willing individuals will proceed to randomisation and allocation of the patient to either the CFT and BPR or control groups.

### Relevant concomitant care permitted or prohibited during the trial *{11d}*

All patients will receive standard oncological care during this trial. These may include active chemotherapy treatments, radiation and/or surgical treatment plans, at the discretion of the treating physicians.

### Provisions for post-trial care *{30}*

All patients, irrespective of group allocation, will receive routine medical management for their cancer recurrence and will be referred for physical therapy on an outpatient basis, or other services, depending on clinical indications and personal needs. All participants that are enrolled into this study are covered by indemnity for negligent harm, through the standard Health Service Executive (HSE) indemnity arrangements. If any participant suffers from any stress or mental health complications arising directly from either intervention during or after the trial, then the participant will be offered further psychological management in line with TAU. This will be offered by a different psychologist that has no direct or indirect involvement with this trial. The research team for this study will liaise with clinical staff attached to the medical teams to make these arrangements.

### Outcomes *{12}*

#### Primary outcome

1. Distress Thermometer Likert scale score [time frame: 3 months after completion of the 6-week group intervention]

The Distress Thermometer (DT) assessment tool will be used to assess for psychological distress levels at the appropriate study time points. This assessment will be conducted by the research assistant. Its scale is measured between ‘0’ and ‘10’, where ‘0’ indicated no distress and ‘10’ indicates extreme distress. A decrease on the DT score of 1 unit (standard deviation of improvement of 2 units) is considered clinically significant and can indicate an improvement in a person’s psychological distress levels [13].

#### Secondary outcomes

Secondary outcomes of the study include levels of depressed mood, levels of anxiety, traumatic distress and reported mental adjustment to cancer. These will be measured using the following questionnaires:

- The Patient Health Questionnaire (PHQ-9) total score [time frame: 3 months post-group programme]
  o The PHQ-9 is a clinically validated, nine-item instrument for screening, monitoring and evaluating symptoms of depression [24]. It incorporates the Diagnostic and Statistics Manual for Mental Disorders, fourth edition (DSM-IV), diagnostic criteria into a brief self-report tool and asks how often a person has ‘been bothered’ by any of the itemised problems in the preceding 2 weeks. Scores range from 0 to 27 and scores ≥ 10 had a sensitivity of 88% and a specificity of 88% for major depression. Scores of 5, 10, 15 and 20 represent mild, moderate, moderately severe and severe depression, respectively.
- The Generalised Anxiety Disorder Questionnaire (GAD-7) score [time frame: 3 months post-group programme]
  o The GAD-7 is an easy-to-use, self-administered screening instrument comprising seven questions. Scores range from 0 to 21, with scores ≥ 10 achieving a sensitivity of 89% and a specificity of 82% for generalised anxiety [Spitzer et al., 2006]. It also enjoys modest effectiveness at screening for other anxiety disorders [25]. Scores of 5, 10 and 15 are the cut-off thresholds for mild, moderate and severe anxiety, respectively.
- The Impact of Event Scale-Revised (IES-R) score [time frame: 3 months post-group programme]
  o The IES-R is a 22-item self-report measure that assesses subjective distress caused by traumatic events [26]. Items correspond to 14 of the 17 DSM-IV symptoms of post-traumatic stress disorder (PTSD). Respondents are asked to name a specific traumatic event with subsequent answers reflecting how distressed or bothered they have felt by each item listed, over the previous 7 days. The instrument yields a score ranging from 0 to 88 along with subscale scores that relate to intrusive phenomena, avoidance and hyperarousal. A cut-off score of ≥ 34 has been suggested for the IER-R as a threshold for probable PTSD, as it has been found to provide good values on sensitivity (86–89%) and specificity (80–81%) in two samples of survivors of war [27].
- The Mini-Mental Adjustment to Cancer Scale (mini-MAC) score [time frame: 3 months post-group programme]
  o The Mini-MAC is a 29-item self-rating questionnaire which was designed to provide a method of assessing specific responses to cancer along the following dimensions: ‘fighting spirit’, ‘helpless/hopeless’, ‘anxious preoccupation’, ‘fatalism’ and ‘avoidance’ [28]. There are published clinical cut-off scores for the dimensions of Summary Negative Adjustment (SNA) and Summary Positive Adjustment (SPA) when scores on thesis scales exceed 36 or fall below 47, respectively [29].

### Participant timeline ***{13}***

The time schedule of enrolment, interventions, assessments and visits for participants are illustrated in Table 2.

**Table 2:** Timeline of participation and evaluation

Outcomes	Instrument	Enrolment Baseline t(0)	6-Wk group intervention	Post-group outcomes t(1)	3-month post-group t(2)
**Primary outcome**					
** Distress Thermometer**	DT	X		X	X
**Secondary outcomes**					
** Depressed** m**ood**	PHQ-9	X		X	X
** Anxiety**	GAD-7	X		X	X
** Traumatic distress**	IES-R	X		X	X
** Mini**-**Mental adjustment to cancer**	Mini-MAC	X		X	X
**Other variables**					
** Patient demographics**		X			

### Sample size *{14}*

Sample size was estimated using a target power of 80%, at a type I error rate of 0.05, and was calculated relative to the primary outcome measure; distress rating on the 11-point Distress Thermometer (DT) scale. The statistical test assumed was an independent samples *t*-test for group differences in the change from baseline to subsequent assessment, assuming that the randomisation ensures no systematic baseline or other covariate group differences. The minimal clinically significant difference for the DT is estimated to be 1 unit (standard deviation of improvement of 2 units), similar to other 11-point Likert scale questionnaires [30]. Calculation produced a suggested sample size of 64 per group. Allowing for a potential attrition rate of 20%, our final sample size is 80 participants per group (8 groups of 10).

### Recruitment *{15}*

Adult patients who are attending the MMUH Cancer outpatient clinics, for the management of cancer recurrence that has occurred since March 2020, who are proficient in the English language will be eligible for consideration. Potentially eligible candidates will be informed of the study at the outpatient clinics of the Cancer and Surgical Directorate, and those expressing initial interest will be provided with a participant information leaflet (PIL) to read. They will also be given a consent form should they wish to participate after reading the PIL.

These individuals will then be followed up with a screening call by the research assistant allied to the research team or if present in the clinic, the research assistant will be on hand to discuss in person. All patients entering the screening phase of the study will be registered on a Study Patient Registration Log and a unique registration number will be assigned. Clinical data will be assessed to evaluate a subject’s eligibility and only those who endorse a score of ≥ 4 on the Distress Thermometer will proceed for study enrolment. Enrolled patients will be evaluated and asked for informed consent, by way of a signed, returned, consent form, after which time they will be randomly allocated to one of two study conditions.

### Assignment of interventions: allocation

#### Sequence generation *{16a}*

A computer-generated randomisation process will be used to allocate the participant to either compassion-focussed therapy and breathing pattern retraining groups or TAU with a 1:1 allocation. It is a feature of the research electronic data capture (REDcap) software being used in this study. The randomisation list will consist of blocks of variable size, with equal allocation to arms within each block.

#### Concealment mechanism *{16b}*

Once baseline data is obtained, it will be entered into research electronic data capture (REDcap), a secure, browser-based web application widely used by researchers for survey data collection. Allocation sequence concealment is a core, automated feature of this software program, which will prevent the participants and researchers from being aware of the generated sequence until after participants are assigned to their groups.

#### Implementation *{16c}*

Patients will be randomised after they are enrolled in the study, having consented and completed the Distress Thermometer (and scored ≥ 4). The research assistant will enter a participant’s baseline data into the research electronic data capture (REDcap) database, a randomisation button will allocate the participant to one of two study arms. Once a participant is randomised, it is not possible to undo this step. After 10–12 participants are enrolled for the interventional group, the psychological group will commence.

### Assignment of interventions: blinding

#### Who will be blinded *{17a}*

This is a non-blinded randomised control trial by virtue of there being one interventional group compared with TAU.

#### Procedure for unblinding if needed *{17b}*

As this is a non-blinded study, an unblinding process will not be necessary.

### Data collection and management

#### Plans for assessment and collection of outcomes *{18a}*

There will be three data-collection time points. Data will be collected via the case record form and self-report questionnaires at baseline (t0), at post-6-week intervention (t1) and at 12-week follow-up (t2). This will be performed entirely by the research assistant. For details of primary and secondary outcome measures, see the ‘Outcomes {12}’ section.

#### Plans to promote participant retention and complete follow-up *{18b}*

During the screening process, study candidates will receive information about the study in the form of a study ‘ad’ and an extensive participant information leaflet describing the study in detail. Study candidates will have ample opportunity to ask questions about the study, at all stages of participation. Eligible candidates will be encouraged to enrol only if they are willing and able to commit to the 18-week study period (6-week intervention plus 12-week follow-up). Every effort will be made by the research assistant to remain in contact, as deemed appropriate, with all study participants throughout their participation. Participants will also have the contact details of the research team should they wish to make contact outside of other study contact points.

#### Data management *{19}*

All data will be recorded on a study-specific case report form (CRF) that has the patient’s unique study identifier code on each page. Study investigators will enter the data from CRFs into a designed study-specific REDCap database that is log-in restricted to formally approved research team members only. Data will be entered primarily by the project’s research assistant (CF) and verified by a different study investigator (SL/DL) to mitigate any risk of data entry errors (e.g. incorrect or duplicate data).

The data collected and all the research-related documents (hard copy CRF, questionnaires and electronic study key) will be stored securely in a locked office in the principal investigator’s office at the Mater Misericordiae University Hospital (MMUH). Only the principal investigator and the co-investigators can have access to these documents. The records will be kept for 5 years following study closure or until such time that study outcomes are fully disseminated. All electronic files will be encrypted and accessed via password-protected computers.

The electronic REDCap database also allows for specified ranges and automatic calculations to reduce entry errors. The study REDCap database will have automatic calculations for study questionnaires and specified ranges entered for each questionnaire response to ensure accurate data entry. Data will be cleaned by investigators upon completion of data collection to ensure good quality data.

#### Confidentiality *{27}*

All the data collected will remain coded and confidential. A unique subject number will be provided to each individual patient participating in the study. The front page of the CRF which will be labelled with the study code number and select patient information will be stored separately to the remainder of the CRF, in the form of an electronic, password-protected study key, containing select data about the participant (such as study code, patient name and DOB) to ensure data is re-identifiable. Only study investigators will have access to the data as CRF forms will be stored in a locked office of the hospital Psycho-Oncology service that only study investigators have access to. CRFs will be retained for the duration of the research project to ensure corresponding REDcap data can be checked and verified.

#### Plans for collection, laboratory evaluation and storage of biological specimens for genetic or molecular analysis in this trial/future use *{33}*

This is not applicable as no biological samples will be collected.

### Statistical methods

#### Statistical methods for primary and secondary outcomes *{20a}*

Outcome analyses will be performed by a dedicated biostatistician. An intention-to-treat analysis will be performed on all randomised patients and a per-protocol analysis will be performed on all patients who complete the study. Descriptive statistics will be calculated for all outcome measures at each time point. Continuous variables will be reported using means, medians, standard deviations and score ranges. Categorical variables will be described using frequencies and percentages.

Descriptive and inferential statistics will be obtained using the appropriate statistical methods that seek to address our identified objectives. The primary analysis will compare the effect of the interventions on the primary outcome; distress levels at 12 weeks post-completion of the intervention period (i.e. c18 weeks post-baseline). All outcome analyses will be conducted according to an intention-to-treat principle, i.e. all randomised participants will be included in the main analysis and will be analysed as randomised, regardless of protocol adherence. Secondary analysis will include the analysis of the primary outcome post-intervention and analysis of the secondary and treatment process outcomes detailed previously post-intervention and at the 12-week follow-up. Linear mixed models on the outcome measures over time will be fitted to evaluate the effect of both study conditions, which intrinsically adjusts for baseline scores.

Statistical significance will be assessed from a *p* value < 0.05 from the group by time interaction term. For all tests, two-sided *p* values will be used, which will be reported to four decimal places with *p* values < 0.001 reported as *p* < 0.001. The Bonferroni method will be used to appropriately adjust the overall level of significance for multiple secondary outcomes as applicable. In the case of a significant result, planned contrasts of the group effects at the post-treatment period and at the 12-week follow-up will be used to investigate the direction and pattern of effects and outlined in advance in the SAP. As a key component of the reporting of the analyses of outcomes, the mean changes (irrespective of statistical significance) and correlations of the measures between the assessment time periods will be obtained. SPSS will be used to conduct the analyses.

### Interim analyses *{21b}*

No interim analyses will be conducted.

### Methods in analysis to handle protocol non-adherence and any statistical methods to handle missing data *{20c}*

Careful attention will be paid to ensure that all participants are fully assessed at all three study time points to minimise missing data. The study’s research assistant will seek to enter hard copy data to the REDcap database in a timely manner, once time point data is collected. This will provide an opportunity for identification of any missing data, which the research assistant will follow up on by encouraging study participants to try and complete any missing answers. We do not plan to use additional statistical methods such as multiple imputation as studies have demonstrated that linear mixed modelling is mostly sufficient to control for missing data [31, 32]. However, multiple imputation will be considered for any measure with over 5% missing data, using a chained equations method robust to non-normally distributed data. This will be fully reported in line with the updated Consolidated Standards of Reporting Trials (CONSORT) recommendations [33].

### Plans to give access to the full protocol, participant-level data and statistical code *{31c}*

The collated data collected by the investigators will be retained for a maximum of 5 years after the analysis has been completed. We will deliver a completely de-identified dataset on appropriate data upon reasonable request and in agreement with the principal investigator and data protection officer.

### Oversight and monitoring

#### Composition of the coordinating centre and trial steering committee *{5d}*

This is a single-centre study. The trial steering committee will consist of the principal investigator (DB), trial coordinator (SL) and personnel responsible for data entry and data management (CF, YOM and DL). This committee will meet monthly to evaluate the progress of the trial, address ongoing organisational and logistical issues and consider any adverse effects. There will be ongoing communication by way of group email for minor or transient needs. More frequent steering committee meetings will be scheduled should the need arise.

### Composition of the data monitoring committee, its role and reporting structure *{21a}*

During the process of obtaining ethical approval for this single-site study, a Data Protection Impact Assessment (DPIA) screening tool was completed. This was analysed by the hospital’s data protection officer (DPO) in MMUH and the DPO of the Host Institution, University College Dublin (UCD). It was deemed that this study posed a low risk to the rights and freedoms of natural persons and therefore a formal DPIA was not needed. Recruitment of participants is expected to be completed within less than 12 months or once the required number of participants needed is fulfilled. Due to the rapid expected inclusion of participants and the known minimal inherited risks associated with this trial, a data monitoring committee was not appointed.

### Adverse event reporting and harms *{22}*

The study interventions associated with this trial do not involve any physical interventions, and all patients will receive standard of care during their study participation period. Therefore, it is not expected that any participants that volunteer to take part in this trial will suffer from any physical complications as a consequence of participation in this study. However, it is possible that some participants may find the study interventions (psychological therapy, mindfulness and breathing) distressing. This is not expected, but in the event anything arises which is out, it will be reported to the principal investigator. The participant will be removed from the trial if merited and further support will be provided.

### Frequency and plans for auditing trial conduct *{23}*

A third-party researcher, affiliated with the UCD clinical research centre, but not involved in the trial in terms of patient recruitment, data collection, data entry and analysis will undertake an auditing process for trial conduct. This will occur every 6 months and will include the following: exploring the REDCap database for accuracy, spot check verification of data entry, screening for duplicate data and ensuring ongoing adherence to data protection guidelines.

### Plans for communicating important protocol amendments to relevant parties (e.g. trial participants, ethical committees) *{25}*

This is not anticipated but should the research group seek to modify any aspect of the research protocol, approval for any such amendments will be sought from the relevant IRB. Participant information leaflets will be updated accordingly and any changes to the published protocol will be reported in full in any future publications.

### Dissemination plans *{31a}*

The intention of the research team is to disseminate the results from this clinical trial in several ways. These will include local talks to various professional audiences at the clinical site of the research project. The plan also includes publication in an international peer-reviewed journal and by way of oral and/or poster presentations at national and international scientific meetings. Positive findings will be reported in full along with potential robust findings of equivalence.

## Discussion

This paper describes the protocol of the COMFORT trial, a RCT comparing the effectiveness of an online, group-based, compassion-focussed therapy (CFT) psychological intervention combined with breathing pattern retraining (BPR), to those receiving standard medical care only, at reducing psychological distress in adults experiencing cancer recurrence during COVID-19. We have endeavoured to enhance the quality of research in the area of hospital-based, psycho-oncology, particularly as it continues to develop in the Irish context. The inclusion criteria for the study are relatively broad, in an effort to capture a representative sample and with the aim of maximising the generalisability of the findings.

There are a number of limitations inherent in this trial, most prominently the inability to blind study participants and clinicians to treatment allocation. We have attempted to address issues of additional biases as far as has been practically possible through the provision of randomisation and concealment of allocation, strategies to minimise and manage incomplete outcome data, the delivery of a specialist combined intervention by an experienced senior psychologist specialising in psycho-oncology and BPR, the recruitment of an adequate sample size and having appropriate duration of follow-up and a priori specification of all primary and secondary outcomes as detailed in this study protocol. We have also traded the reported downside of a virtual group (reduced social interaction with and support from fellow peers) off against this platform removing a potential geographical boundary for some individuals unable to commute to Dublin on a regular basis.

To our knowledge, this will be the first RCT to assess the effectiveness of a combined psychological and breathing pattern retraining intervention for cancer recurrence. The study results will add to current knowledge in the field of psycho-oncology and general cancer management and will have the potential to inform the delivery of best-practice treatments for cancer patients.

### Trial status

The trial is registered on ClinicalTrials.gov Identifier: NCT05518591. The current protocol is version 2 of 05 February 2021. Participant recruitment began on 25 August 2022, and full patient recruitment is estimated to be completed by September 2023. Currently, 8.5% of patients have been recruited.

ClinicalTrials.gov. Registered on 25 August 2022. All items from the World Health Organization Trial Registration Data set have been included.

## Data Availability

The final trial dataset generated from this study will be made available from the corresponding, first and/or principal authors upon reasonable request.

## Abbreviations

FOR: Fear of recurrence
MMUH: Mater Misericordiae University Hospital
NCCN: National Comprehensive Cancer Network
DT: Distress Thermometer
CFT: Compassion-focussed therapy
CBT: Cognitive behavioural therapy
BPR: Breathing pattern retraining
RCT: Randomised control trial
HSE: Health Service Executive
PHQ: Patient Health Questionnaire
DSM-IV: Diagnostic and Statistics Manual, Fourth Edition
GAD: Generalised anxiety disorder
IES-R: The Impact of Event Scale-Revised
Mini-MAC: Mini-Mental Adjustment to Cancer
PIL: Participant information leaflet
REDcap: Research electronic data capture
CRF: Case report form
SAP: Statistical Analysis Plan
CONSORT: Consolidated Standards of Reporting Trials
DPIA: Data Protection Impact Assessment
DPO: Data protection officer
UCD: University College Dublin
